# A Novel Algorithm for the Precise Calculation of the Maximal Information Coefficient

**DOI:** 10.1038/srep06662

**Published:** 2014-10-17

**Authors:** Yi Zhang, Shili Jia, Haiyun Huang, Jiqing Qiu, Changjie Zhou

**Affiliations:** 1 Department of Mathematics, Hebei University of Science and Technology/Hebei Province Key Laboratory of Molecular Chemistry for Drug, Shijiazhuang, Hebei 050018, China; 2 Department of Information Retrieval of Library, Hebei University of Science and Technology, Shijiazhuang, Hebei 050018, China

## Abstract

Measuring associations is an important scientific task. A novel measurement method *maximal information coefficient* (MIC) was proposed to identify a broad class of associations. As foreseen by its authors, MIC implementation algorithm ApproxMaxMI is not always convergent to real MIC values. An algorithm called SG (Simulated annealing and Genetic) was developed to facilitate the optimal calculation of MIC, and the convergence of SG was proved based on Markov theory. When run on fruit fly data set including 1,000,000 pairs of gene expression profiles, the mean squared difference between SG and the exhaustive algorithm is 0.00075499, compared with 0.1834 in the case of ApproxMaxMI. The software SGMIC and its manual are freely available at http://lxy.depart.hebust.edu.cn/SGMIC/SGMIC.htm.

All kinds of relationships determine the development of things^1^,^2^,^3^. Relationships and associations should therefore be identified and measured to explore the rules of development. A typical example is measuring the relationships between genes by determining the associations between their expression profiles^4^,^5^. Many methods have been developed to measure associations through calculation of correlation coefficients, such as Pearson's, Spearman's, mutual information^6^,^7^, CorGC^8^, and maximal correlation^9^. Recently, Reshef *et al.*^10^ proposed a novel correlation measurement “maximal information coefficient” (MIC), and gave a 1-D dynamic programming algorithm, ApproxMaxMI, to calculate MIC. MIC does not rely on the distributional assumptions of measured data and could identify a broad class of associations compared with previous studies. The MIC of two vectors *x* and *y* is defined as follows.


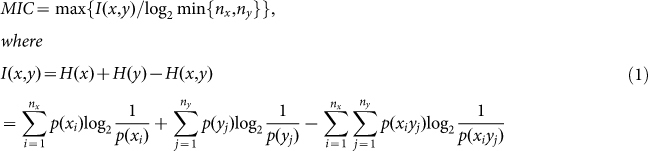
*n_x_***·***n_y_*<*B*(*n*), *B*(*n*) = *n*^0.6^. In calculating MIC for gene expression profile vectors *x* and *y*, *n* is the number of data points of gene expression profiles, and *n_x_*, *n_y_* is the number of bins of the partition of the *x*- and *y*-axis^10^, respectively. After MIC and its algorithm were published, many applications and discussions appeared^11^,^12^,^13^,^14^,^15^. Reshef *et al.* also foresaw the possible disadvantage of the algorithm “ApproxMaxMI”, and suggested it should be replaced in the future if a method efficiently ﬁnds solutions that are closer to optimal or even optimal is developed^10^. Here, we conducted an initial attempt in this direction.

## Results

### ApproxMaxMI does not optimize the partition of the *y*-axis

**Figure 1:**
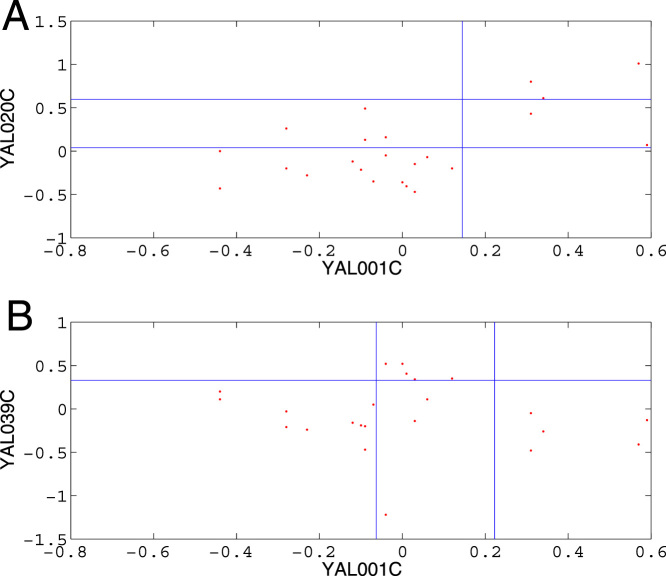
The optimal grid for MIC. (A, B): optimal *x*-by-*y* grid of gene pairs *YAL001C*:*YAL020C* and *YAL001C*:*YAL039C* obtained by the exhaustive algorithm. Each subfigure includes 23 discrete points.

ApproxMaxMI first fixes an equipartition of *n* data points with horizontal lines, and then calculates MIC values by moving vertical lines to optimize the *x-axis* partition. However, the partition of the *y*-axis should be optimized simultaneously instead of being fixed as an equipartition. MIC value of a pair of gene expression profiles (*YAL001C:YAL020C*) of yeast^16^ was 0.30732 according to ApproxMaxMI, but the MIC value optimized by exhaustive algorithm was 0.51582, this value can be obtained directly from the partition scheme in Fig. 1A. Similarly, the MIC value of *YAL001C:YAL039C* was 0.28519 according to ApproxMaxMI, but the MIC value optimized by exhaustive algorithm was 0.42340 which can be obtained directly from the partition scheme in Fig. 1B. Such significant differences can also be identified from the gene expression profiles of fruit fly (Fig. S1–S4; Droso174figure.zip at http://lxy.depart.hebust.edu.cn/SGMIC/SGMIC.htm). From the observations, as foreseen by Reshef *et al*., the MIC values calculated by ApproxMaxMI were not always convergent to optimum MIC values for the following simple proposition:

Proposition 1.1: Fixing an equipartition of *n* data points by horizontal lines is neither a sufficient nor a necessary condition to obtain MIC = max{*I*(*x, y*)/log_2_min{*n_x_*, *n_y_*}}.

Proposition 1.1 is proven in Supplementary Section 2.1. In this sense, ApproxMaxMI does not achieve the equivalent transformation from a 2-D search to a 1-D search. Theorem 1 and Proposition 6.12 of Reshef *et al*^10^ are related to algorithm convergence of ApproxMaxMI, however they only discuss the convergence to 0 of the MIC value of independent *X* and *Y* as the number of data points *n*→∞ rather than the convergence to the global optimum MIC value. The convergence of ApproxMaxMI cannot be proven by the approaches because of the following proposition:

Proposition 1.2: The limit property of a series is not equivalent to the property of the series items. For example, 
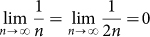
 but 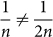 when *n* is finite.

### Flow and Theory of SG

Based on Simulated annealing^17^,^18^ and Genetic algorithms^19^,^20^, we proposed a novel algorithm called SG to calculate MIC values. This algorithm was implemented in SGMIC software (Algorithms 1 and 2 of Supplementary Section 2.2). The convergence of SG is proven according to Propositions 1.3 to 1.6, which are shown below. The proof of Propositions 1.3 to 1.6 is shown in Supplementary Section 2.3 to 2.6.

Proposition 1.3: If the transition matrix *P* of SG is derived from proportional selection, mutation probability 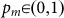, crossover probability 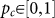, and simulation annealing 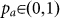
17,18, then *P* = SCMA is primitive.

Proposition 1.4: In the SG algorithm, initial state *i* can be transited into any state *j* in finite expected transition time.

It's worth noting that this proposition guarantees that SG can reach the state corresponding to the optimum MIC values in finite steps from any given initial state. In fact, SG can converge to the global optimum of MIC values shown as follows.

Proposition 1.5: SG is convergent.

Proposition 1.6: SG is equivalent to the exhaustive algorithm with a sufficient number of iterations.

The equivalence of SG and the exhaustive algorithm is shown in Table S1 of Supplementary Section 3.1.

To set a gold standard for these algorithms, we provide an exhaustive algorithm (Algorithm 3 of Supplementary Section 2.2) to calculate MIC values. The flow chart of SG (Fig. S5) is shown in Supplementary Section 3.2.

### Optimizing the Calculation of MIC Values with SG

**Figure 2:**
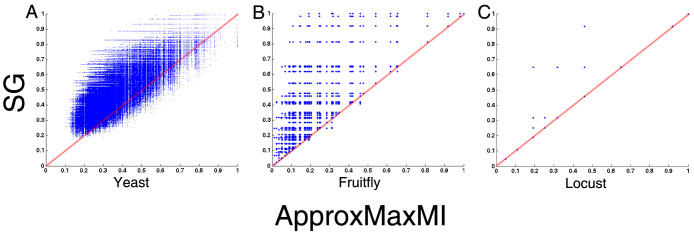
Comparison of MIC values between SG and ApproxMaxMI for three species. The solid line represents the function y = x. (A–C): The MIC value comparison of the two algorithms for yeast, fruitfly and locust.

SG can optimize the calculation of MIC values. Most MIC values from SG were much larger than those from ApproxMaxMI (Fig. 2 and Table S2 of Supplementary Section 3.3.) To compare the performance of SG with that of ApproxMaxMI, we employed the exhaustive algorithm to calculate 1,000,000 MIC values for fruit fly. There are 999,807 relationships having MIC values from SG and the exhaustive algorithm that matched. However, the match ratio of ApproxMaxMI was only 24,478/1,000,000 = 2.4%. Two-tailed *t* test showed that the average of the MIC values by SG (0.4787) was significantly different from that by ApproxMaxMI (0.3417), with a p-value of 10^−100^. Moreover, the mean squared difference between SG and the exhaustive algorithm is 0.00075499, compared with 0.1834 in the case of ApproxMaxMI. Therefore, SG can optimize the calculation of MIC values more effectively than ApproxMaxMI (Fig. S6 of Supplementary Section 3.4). The exhaustive algorithm is very time-consuming with more time points. It takes four and a half hours to calculate an MIC value for yeast (23 time points), so we only calculated the MIC values for *YAL001C*:*YAL020C* and *YAL001C*:*YAL039C* with the use of the exhaustive algorithm (Fig. 1A and 1B). We also compared SG with ApproxMaxMI on a larger data set of three species. From the 4,498,500 relationships mentioned above in the 3,000 genes of yeast^16^, fruit fly^21^, and locust^22^, our SG algorithm can obtain 4,348,253, 4,377,054, and 4,046,571 MIC values (http://lxy.depart.hebust.edu.cn/SGMIC/SGMIC.htm), respectively, which are much larger than those of ApproxMaxMI. Therefore, SG can calculate MIC values more optimal than ApproxMaxMI.

For random clouds at sample size *n*, the larger the value of *n* is, the more individuals (or chromosomes) and longer running time are needed to show the advantage of SG method over ApproxMaxMI. Specifically, for n < 30 points, 20 individuals are usually enough; for 30 < n < 50, 100 individuals are needed; for 50 < n < 100, we need 1000 individuals; while for 100 < n < 200, at least 10000 individuals are required. These data and running time are available in file populationchangewithN.xlsx (http://lxy.depart.hebust.edu.cn/SGMIC/SGMIC.htm). For 200 < n < 500, perhaps over 100000 individuals are needed. In this sense, for gene expression profile data, which usually have less than 50 time points, 100 individuals are enough. Because computing MIC value by SG for 500 random points need over 100000 individuals and over 12 hours, we can only calculate a SGMIC value for an example in points500example.xlsx (http://lxy.depart.hebust.edu.cn/SGMIC/SGMIC.htm), and the MIC value by SG with 100000 individuals is 0.150593, while the MIC by ApproxMaxMI is 0.14799.

For non-random relationships, the efficiency of SG is good, as shown in next section, where the majority of MIC values by SG were near 1 when the relationships had no noise, although only 20 individuals are used in SG.

## Discussion

### Properties of MIC Values by SG

**Figure 3:**
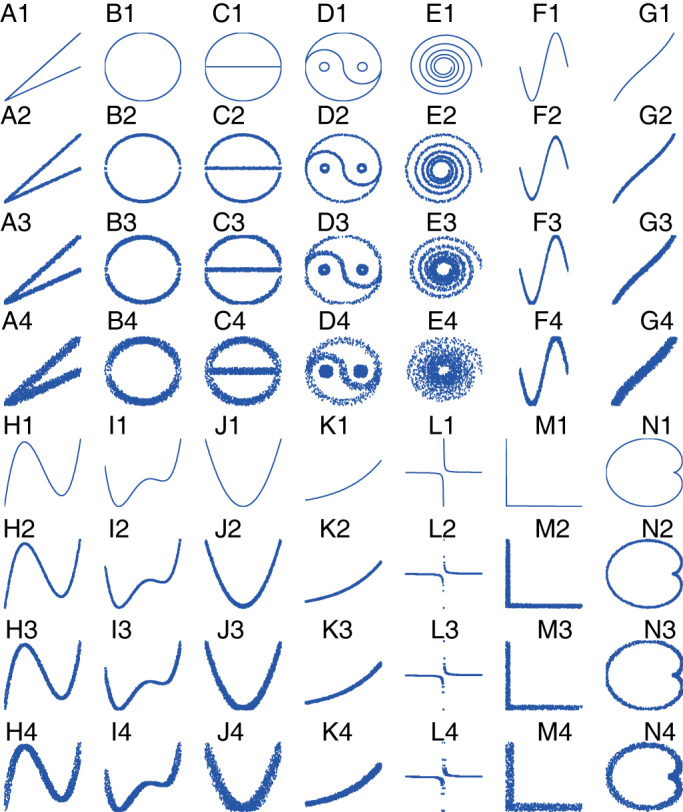
14 figures of representative relationships without noise and their 42 figures with noise. In calculating correlation, 2,000 points are randomly selected from each figure.

Because SG is a precise method in calculating MIC values, we redescribed the main properties of MIC values for four classes of main relationships, namely, function relationship, non-function relationship, function with noise, and non-function with noise. Fourteen relationships without noise were drawn by us in Fig. 3, and their formulas are presented in Table S4 of Supplementary Section 4. Function relations are represented by trigonometric functions (e.g., sin and tan), power functions (e.g., *y* = *x*^2^), exponential functions (e.g., *y* = 10*^x^*), inverse proportion functions (e.g., 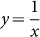), and composite functions. Meanwhile, some non-function relationships, such as the taijitu (symbol for the yin-yang principle, which originated from Yi Jing of ancient China), galaxy figure, heart-shaped line, and polygonal line, were also selected. To investigate the effect of noise on relation-measure algorithms, three relationships with increasing noise for each of the 14 relationships were drawn by us in Fig. 3. To compare different algorithms, we calculated MIC values by SG, mutual information through Covshrink-KPM (*Hausser, J., Strimmer, K. Entropy inference and the James-Stein estimator, with application to nonlinear gene association networks. J. Mach. Learn. Res. ****10****, 1469–1484 (2009)*), maximal correlation^10^ (ACE), Spearman's, Pearson's, and distance correlation (*RIZZO, M. L., SZÉKELY, G. J. Energy: E-statistics (energy statistics). R package version 1.1-0 (2008)*) for all 56 figures. Then we calculated these correlations with 2,000 random points from each figure. We came to the following conclusions after analyzing the results of the calculations (Table S5 of Supplementary Section 5):

First, the majority of MIC values by SG were near their full score of 1 when the relationships had no noise, although only 20 individuals are used in GA, it confirmed the efficiency of SG in computing MIC values. However, when some relationships (e.g., Fig. 3 A–E) were involved, SG and maximal correlation (ACE) algorithm exhibited a much better performance than that of ApproxMaxMI. Second, the MIC values by SG strictly decreased with increasing noise, a finding indicating that SG has a strong ability to distinguish noise from real signals. However, a strict decreasing trend was not observed with other algorithms. For example, the maximal correlation (ACE) of d1, d2, d3, and d4 was 0.33508, 0.64533, 0.55032, and 0.22122, respectively.

### The Application in Predicting Yeast Protein Interaction

**Figure 4:**
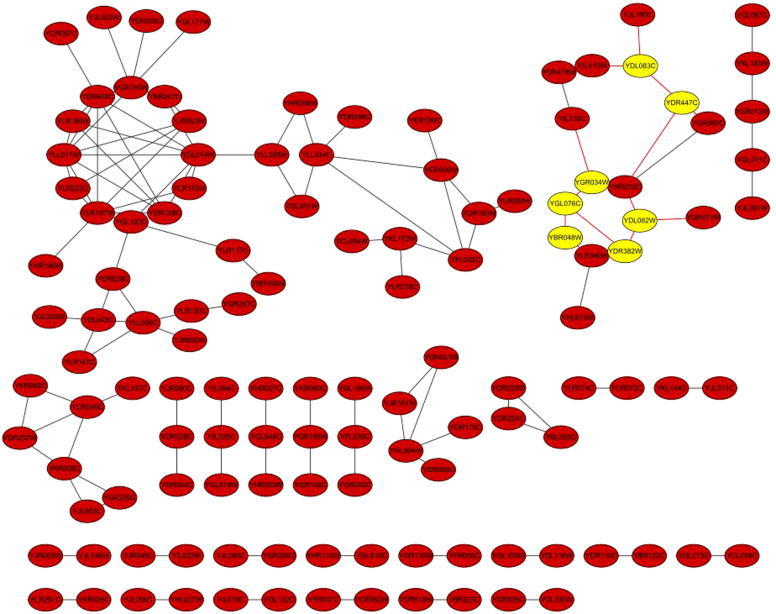
The 125 interactions of yeast proteins with high SG value and low ApproxMaxMI values. They are not included in the DRYGIN database but appear in CCSB interactome. The associations between genes in yellow circles have very low MIC values by ApproxMaxMI but high MIC values by SG.

To describe the performance of SG more intuitively, we use the Data Repository Yeast Genetic Interactions (DRYGIN for short)^23^, which are derived from large-scale Synthetic Genetic Array (SGA) genetic analysis and the Genetic interaction score (ε) can represent the genetic interaction quite accurately. The file sgadata_costanzo2009_intermediateCutoff_101120.txt.gz contains 76406 genetic interactions with an intermediate cutoff applied (|ε| > 0.08, p-value < 0.05). The MIC value of 96.46% (73702 out of 76406) genetic interaction by SG is larger than that by ApproxMaxMI, though the maximum MIC values by both methods are the same, i.e. 0.9986. We found 125 interactions (Fig. 4) with high SG score but low ApproxMaxMI, these interactions are not included in the DRYGIN but appear in CCSB interactome^24^. For example, the ApproxMaxMI values of interactions YDR382W and YDL082W, YDR447C and YDL083C, YGL076C and YBR048W, YGR034W and YGL076C are 0.54236, 0.56215, 0.52781 and 0.57648 respectively, while the SG values are all 0.932112. The four interactions are all verified by CCSB interactome but are not included in DRYGIN, which shows the advantage of SG over ApproxMaxMI.

## Methods

### Datasets

The gene expression profile datasets used are from transcriptome of yeast^16^, fruit fly^21^ and locust^22^. These datasets also can be downloaded at http://lxy.depart.hebust.edu.cn/SGMIC/SGMIC.htm

### Simulated Annealing and Genetic Algorithm in SG

Simulation annealing algorithm^17^,^18^ is used in the process of genetic algorithm, and the simulation annealing can enhance the optimization result of GA. For example, in calculating MIC values for 1,225 pairs of vectors consisting of 200 random numbers, simulation annealing increases MIC values of 762 pairs of vectors (as shown in Table S6 in supplementary section 6 and file needsimulationanneal.xlsx in http://lxy.depart.hebust.edu.cn/SGMIC/SGMIC.htm). We used a multithread method to calculate MIC values with the use of genetic algorithm^19^,^20^. In each thread, the number of chromosomes is 20 by default. Each chromosome consists of genes, which are the abscissa (*xgene*) of vertical partition lines and the ordinate (*ygene*) of horizontal partition lines, and these genes form an *x*-by-*y* grid. We crossed over an *xgene* only with another *xgene* and a *ygene* only with another *ygene* during the genetic crossover step. After the mutation, crossover, and simulation annealing, we calculated the fitness of each chromosome and kept the optimum solution. In the annealing, the reproduction operator was derived from Metropolis criteria. The fitness value, championed for over 30 generations, was considered as the MIC value from SG.

### Exhaustive Algorithm

Inputting the expression profiles of a pair of genes, we transformed them into a consecutive integer series, kept the order relation of the original *x* and *y* coordinates, and denoted the maximum number of the series as *n*. The computation loop for the *x* and *y* coordinates was 1 to 2^n-1^-1. For both *x* and *y* coordinates, we transformed the number of the current loop into binary number. Using the binary number, we determined whether to insert a partition line between two space-adjacent expression points (1 indicates a partition line and 0 means no partition line). MIC values were saved for each loop out of (2^n-1^-1)^2^ loops, and the maximum value was considered as the MIC value by the exhaustive algorithm.

### Mutation Procedure

The mutation with self-adapting parameters is used, and the mutation frequency is defined as: 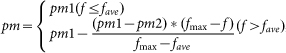, where *f*_max_ is the largest fitness of current population, *f*_ave_ is the average fitness of current population, *f* is the individual fitness. Parameters pm1 > pm2 with 0 < pm1,pm2 < 1 are mutation parameters. First, an individual (or chromosome) is assigned a random number in (0,1): if the random number is no less than *pm*, then the individual will mutate; skip the step otherwise. Here, an individual (or chromosome) is a set of vertical or horizontal lines constituting the bins of the partition of the *x*- and *y*-axis^10^. Mutating an individual is changing the positions of some vertical or horizontal lines along the *x*- or *y*-axis. The first and last horizontal (or vertical) lines are only allowed to move between its original position and the next line, and the other lines are allowed to move between its left and right neighbor lines freely.

### P-value of SG algorithm

We use a non-parameter test method: Wilcoxon Rank-sum Test to calculate the p value, the Rank-sum Test is capable of determining if two gene profiles are independent. The p-value can be seen from file Pvalue.xlsx in http://lxy.depart.hebust.edu.cn/SGMIC/SGMIC.htm.

### The optimization of B(n)

The setting *n_x_**·**n_y_* < *B*(*n*), *B*(*n*) = *n*^0.6^ is mainly based on the following consideration. Based on the Theorems 1 and 2 in Section 6.2 of Reshef *et al.*^10^, we know that setting B(n) < n can avoid inﬂated MIC scores with large B(n). Then we search for an optimal value of B(n), above which statistically independent data receive scores bounded away from zero as sample size grows, and below which they receive scores approaching zero. The Supplementary Section 7 Fig. S7 shows the *alpha* = 0.6 meets the criterion.

## Author Contributions

Y.Z. designed and proved the algorithms, analyzed the data, and wrote the article. S.J. programmed the SGMIC software, drew the figures, and analyzed the data. H.H., J.Q. and C.Z analyzed the data and wrote the article. All the authors reviewed the manuscript.

## Supplementary Material

Supplementary Information supplimentary material
