# Supplementary material for: A Novel Algorithm for the Precise Calculation of the Maximal Information Coefficient
Source: Sci Rep. 2014 Oct 17;4:6662. doi: 10.1038/srep06662 (PMC4200418; doi:10.1038/srep06662)
Supplement: Supplementary Information — supplimentary material [file srep06662-s1.doc]

**Supporting Online Material for**

**A Novel Algorithm for the Precise Calculation of the Maximal Information Coefficient**

Yi Zhang^1*^, Shili Jia^1^, Haiyun Huang^2^ , Jiqing Qiu^1^,Changjie Zhou^1^

^1^Department of Mathematics, Hebei University of Science and Technology/ Hebei Province Key Laboratory of Molecular Chemistry for Drug, Shijiazhuang, Hebei 050018, China

^2^Department of Information Retrieval of Library, Hebei University of Science and Technology, Shijiazhuang, Hebei 050018, China

*To whom correspondence should be addressed: zhaqi1972@163.com

**Guide**

Section 1 : Fig. S1 to S4

Section 2: proof of propositions1.1-1.6. Pseudo codes for SG and Exhaustive algorithm

Section 3: Table S1and S2, the flow of SG, and Fig.S5, S6.

Section 4: Table S4

Section 5: Table S5

Section 6: Table S6

Section 7: Fig. S7

Other Supplementary materials are available from “<http://lxy.depart.hebust.edu.cn/SGMIC/SGMIC.htm>” :

1. Droso174figure.zip. In each figure, there is a correct MIC value of a pair of gene expression profiles of fruit fly from Exhaustive algorithm and its corresponding x-by-y grid. For comparison, the MIC values by ApproxMaxMI are shown.
2. G3000.csv, F3000.csv, Y3000.csv. The 3,000 gene expression profiles from locust, fruit fly and yeast, respectively.
3. compare_locust.rar, compare_fruitfly.rar, compare_yeast.rar. The 4,498,500 MIC values obtained from SG and ApproxMaxMI based on 3,000 genes of locust, fruitfly and yeast, respectively
4. Exhaustive-SG-ApproxMaxMI.rar. It includes MIC values of 1,000,000 pairs of gene expression profiles of fruity fly using Exhaustive algorithm, SG and ApproxMaxMI, respectively.
5. Pvalue.xlsx. MIC values and p-value by SG for 1,225 pairs of vectors consisting of 100 random numbers.
6. needsimulationanneal.xlsx. In calculating MIC values for 1,225 pairs of vectors consisting of 200 random numbers, simulation annealing can increase MIC values of 762 pairs of vectors
7. points500example.xlsx. MIC values of 500 random points by SG and ApproxMaxMI.
8. populationchangewithN.xlsx. For 30, 50, 100, 200 or more random points, SG need increase the number of individuals.

**Section 1**

1. **FigS1-S4:** four relationships of fruit fly gene expression profiles and their corresponding x-by-y grid of MIC values. It is shown that the MIC values by ApproxMaxMI are less than those by Exhaustive algorithm.


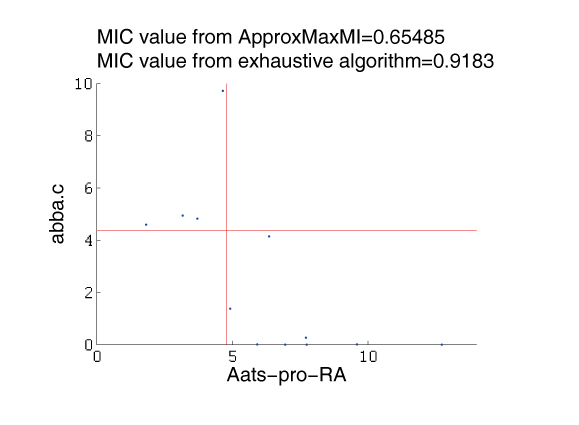

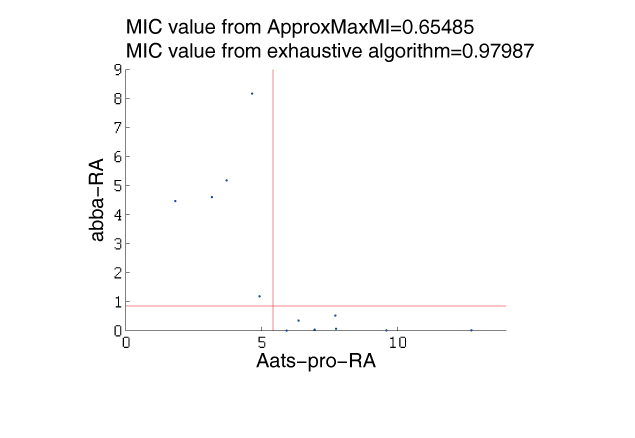

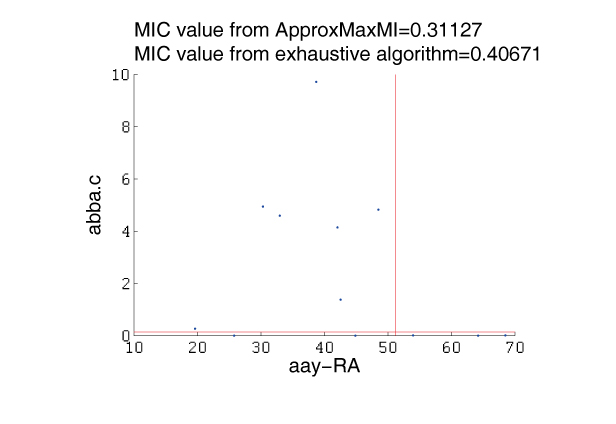

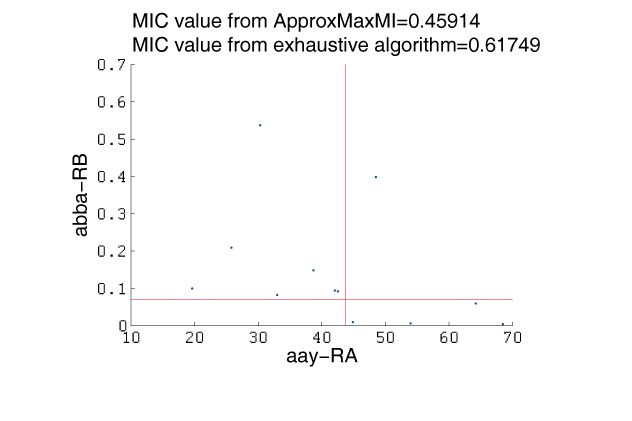


**Section 2**

**2.1**

Proposition 1.1: Fixing an equipartition of *n* data points by horizontal lines is neither a sufficient nor a necessary condition to obtain$\text{MIC}=\max\{I(x,y)/\log_{2}\min\{n_{x},n_{y}\}\}$.

*Proof.* As shown in the two examples in Fig.1A,1B, the data points of neither example are equally partitioned by horizontal lines, and the corresponding maximum values $\text{MIC}=\max\{I(x,y)/\log_{2}\min\{n_{x},n_{y}\}\}$obtained from Exhaustive algorithm are larger than the values calculated by ApproxMaxMI, which are derived from y-axis equipartition. Thus, equipartition of *y*-axis does not lead to accurate maximum of$I(x,y)/\log_{2}(\min\{n_{x},n_{y}\})$sufficiently, and the maximum does not necessarily come from the equipartition of *y*-axis. #

**2.2: Pseudo codes for SG and Exhaustive algorithm**

**_________________________________________________________________**

Algorithm 1: SG

___________________________________________________________________________-

Require: the points from dataset in a csv file.

Ensure: return the MIC value from SG.

Main thread:

1: read point

2: save point in a struct;

3: for i=0 to num_row

4: for j=i+1 to num_row

5: for m=1 to num_point

6: for n=1 to num_point

7: if (m+1)*(n+1)<num_point^0.6

8: take struct , m and n into newthread;

9: end if

10: end for

11: end for

12: get S from every newthread;

13: MIC by SG =max[S]

14: end for

15: end for

__________________________________________________________________________

___________________________________________________________

Algorithm 2: newthread GA

*______________________________________________________________*

Require: struct, including the position of point;

Require: *m* and *n*, they represent the number of bins of partition of x-axis and y-axis respectively， and$fitness=I(x,y)/\log_{2}\min\{n_{x},n_{y}\}$.

Ensure: return value *S* based on the *m,n*

newthread GA:

1: random x-axis,y-axis partition based on m,n;

2: random chromosome;

3: Initialization:

4: S=first maximum fitness value;

5: P=0;

6: End initialization.

7: Optimized was reserved;

8: Select operation:

9: The selected probability$P_{i}=\frac{F_{i}}{\sum_{i=1}^{M} F_{i}}(i=1,2,\cdot\cdot\cdot,M)$

10: Crossover operation:

11: Arithmetical Crossover: $\alpha_{i}$=random[0,1]

12: Mutation operation

13: Optimized was reserved;

14: Simulated Annealing;

15: S1= maximum fitness value;

16: If S>S1

17: P=p+1;

18: Else

19: S=S1;

20: P=0;

21: end if

22: If p>30

23: Return S;

24: else

25： Goto:7:

____________________________________________________________________________

______________________________________________________________________________

Algorithm3 Exhaustive

*______________________________________________________________________________-*

Require: the data points from dataset in a csv file.

Ensure: return the exhaustive value of MIC for all possible combinations of partition of x-axis and y-axis

Main thread:

1: read point

2: save point in a struct;

3: for i=0 to group

4: for j=i+1 to group

5: for m=1 to 2^(num_point-1)

6: for n=1 to 2^(num_point-1)

7: m to binary a[];

8: n to binary b[];

9: the num of 1 in a[] is cx;

10: the num of 1 in b[] is cy;

11: if (cx+1)*(cy+1)<num_point^0.6

12: the position of 1 in a[] and b[] represent the position of partition line.

13: end if

14: end for

15: end for

16: get S for each pair of m and n

17: MIC by Exhaustive algorithm=max[S]

18: end for

19: end for

____________________________________________________________________________-

**2.3** Definition of the Genetic Algorithm^19,20^

The genetic algorithm (GA) is used to solve optimization problems such as: $\max\{f(b)|b\in B^{l}\}$, here $0<f(b)<\infty$for all $b\in B^{l}=\{0,1\}^{l}$and $f(b)\neq const$. A standard GA includes the following steps:

1) Generating an initial population:$b_{1}^{1},b_{2}^{1},\cdots,b_{n}^{1}$.

2) Calculating the fitness for individuals: $f(b_{1}^{1}),f(b_{2}^{1}),\cdots,f(b_{n}^{1})$

3) Selecting individuals based on the fitness: $p(b_{i}^{1})=\frac{f(b_{i}^{1})}{\sum_{j=1}^{n} f(b_{j}^{1})}$

4) Do following iterations until stopping criterions are satisfied

4.1) Crossover individuals

4.2) Mutate individual $b_{i}\to b_{j}$at step *t* based on the *transition probability*:$M_{ij}(t)=p_{m}^{H(b_{i},b_{j})}(1-p_{m})^{l-H(b_{i},b_{j})}$, *H* is hamming distance.

4.3) Calculate the fitness for individuals

4.4) Select individuals based on the fitness

**2.4 Finite Markov chains**

The process calculating a MIC from SG is a finite process, and it can be described by a finite Markov chain, which is a probabilistic trajectory over a finite state space *S* of |*S*|=*n*. The transition probabilistic matrix $P_{n\times n}$ describes the transition probabilities between *n* states. The Markov chain is called *homogeneous*, if the *P* is independent of step *t*. The matrix *P* can be classified into following classes:

(1) *nonnegative*, if all$P_{ij}\geq0$; *positive*, if all$P_{ij}>0$.

(2) *primitive* or *ergodic*, if there exists a *k* which is a positive integer, $P^{k}$is positive.

(3) *reducible*, if symmetric permutation can transform positive *P* into lower triangular block matrix; otherwise, it is called *irreducible*.

(4) *stochastic*, if $\sum_{j=1}^{n} P_{ij}=1$and $P_{ij}\geq0$, for $i\in\{1,2,\cdots,n\}$.

(5) *stable*, if *P* is *stochastic* and has identical rows.

(6) *column allowable*, if *P* is *stochastic* and has at least one positive entry in each column. Similarly, we can define *row allowable.*

(6) *row allowable*, if *P* is *stochastic* and has at least one positive entry in each row*.*

LEMMA 1^25^

Let $P_{n\times n}$ be a reducible stochastic matrix. Then there exists a primitive stochastic matrix $D_{m\times m}$ and$R,T\neq0$, such that is a stable stochastic matrix with, where is unique regardless of the initial distribution, and satisfies: for $1\leq i\leq m$and for$m<i\leq n$. #

Briefly, when, $P^{k}$can converge to a unique matrix $P^{\infty}=\left\{\begin{matrix} \pi\\ \vdots\\ \pi\end{matrix}\right\},\pi=(p_{1},p_{2},\cdots,p_{m},0,\cdots,0),p_{i}>0,i=1,\cdots,m.\sum_{i-1}^{m} p_{i}=1.$

**2.5 SG is ergodic.**

Proposition 1.3: If the transition matrix *P* of SG is derived from proportional selection, mutation probability$p_{m}\in(0,1)$, crossover probability$p_{c}\in[0,1]$, and simulation annealing $p_{a}\in(0,1)$^17,18^, then *P* = SCMA is primitive.

*Proof.*

The crossover operator may be regarded as a random total function whose domain and range are *S*, i.e., each state of *S* is mapped probabilistically to another state. The transition matrix of the Crossover operator is denoted by C, therefore, $\sum_{j=1}^{n} \text{C}_{ij}=1$and$\text{C}_{ij}\geq0$, for $i\in\{1,2,\cdots,n\}$. To say, C is a stochastic matrix. For mutation matrix M, each$M_{ij}(t)=p_{m}^{H(b_{i},b_{j})}(1-p_{m})^{l-H(b_{i},b_{j})}$>0, M is a positive matrix. Let D=CM, by matrix multiplication $d_{ij}=\sum_{k=1}^{n} c_{ik}.m_{kj}>0.$Hence CM is positive. A simulation annealing matrix A can be regarded as a kind of mutation matrixes, so A is a positive matrix. Obviously, E=CMA is positive.

The probability that selection does not alter the state generated by mutation，and in matrix S, at least we have $s_{ii}\geq\frac{\prod_{k=1}^{n} f(\pi_{k}(i))}{\left(\sum_{j=1}^{n} f(\pi_{k}(i))\right)^{n}}>0$, for all $i\in S$, i.e., in each row of matrix S, we have at least one element is positive. Because P=SCMA=SE, $p_{ij}=\sum_{k=1}^{n} s_{ik}.e_{kj}>0.$ P=SCMA is positive, also primitive and ergodic. #

Remark: This is the theoretical basis of our SG algorithm.

Proposition 1.4: In the SG algorithm, initial state *i* can be transited into any state *j* in finite expected transition time.

*Proof*: In the SG algorithm, the markov transition matrix P=SCMA is primitive, hence the markov chain is ergodic. Based on the conclusions in the literature^25^, we can come to the conclusion obviously. #

**2.6 SG converges to its global optimum**

In optimization theory, an algorithm is said to be *converge* to the global optimum if it generates a sequence of solutions or function values in which the global optimum is a limit value. Obviously, ApproxMaxMI transformed a 2D search to a simplified 1D search, cannot guarantee to converge to the global optimum MIC. Based on the markov chain theory, Eigen^26^ proved that, GA has probabilistic convergence of the best solution within a population to the global optimum under *elitist selection* (the best individual survives with probability 1). Rudolph^25^ proved that, recording the best individuals for each iteration, the GA also converges to its optimum. However, our algorithm SG combines the GA with SA (simulation annealing), and its convergence has not been proved before. Here, we will give the following detailed process proving the convergence of SG as follows.

*After* selecting, crossover and mutation, SG calculates the fitness f(x_i_) for each chromosome, keeping the optimum chromosome. It can be regarded as to keeping a super individual in each step and the super one does not take part in the evolutionary process, and hence enlarge the state number to $2^{(n+1)l}$from$2^{nl}$. Different populations including *n* individuals correspond to different states. $\pi_{k}(i)$is the *k*-th individual of *i*-th population. For convenience, we take the super one as the *0*-th individual in *i*-th population $\pi_{0}(i)$.

(1) For the $2^{(n+1)l}$ states, the state transition matrixes corresponding to crossover, mutation, selection and simulation annealing are $S^{+},C^{+},M^{+},A^{+}$respectively, where

$S^{+}=\left(\begin{matrix} S & & & \\ & S & & \\ & & \ddots& \\ & & & S \end{matrix}\right),C^{+}=\left(\begin{matrix} C & & & \\ & C & & \\ & & \ddots& \\ & & & C \end{matrix}\right),M^{+}=\left(\begin{matrix} M & & & \\ & M & & \\ & & \ddots& \\ & & & M \end{matrix}\right),A^{+}=\left(\begin{matrix} A & & & \\ & A & & \\ & & \ddots& \\ & & & A \end{matrix}\right).$ $S^{+}C^{+}M^{+}A^{+}\text{=}\left(\begin{matrix} \text{SCMA} & & & \\ & \text{SCMA} & & \\ & & \ddots& \\ & & & \text{SCMA} \end{matrix}\right),$where SCMA is positive based on above Proposition 1.3. The copy operation is represented by an upgrade matrix U, its rank is $2^{(n+1)l}$, and it upgrades an intermediate state containing an individual better than its super individual to a state where the super individual equals to the better individual. Let $b=\mathrm{argmax}\{f(\pi_{k}(i))|k=1,\cdots,n\}\in B^{l}$denotes the best individual of the population at any state *i* excluding the super individual. Then$u_{ij}=1$,$u_{ir}=0,r\neq j$, $j=(b,\pi_{1}(i),\pi_{2}(i),\cdots,\pi_{n}(i))\in S,$if $f(\pi_{0}(i))<f(b),$and undergone state upgrade$i\to j$. Otherwise, when$f(\pi_{0}(i))\geq f(b),$undergone state upgrade$i\to i$,$u_{ii}=1$, $u_{ir}=0,r\neq i.$

(2) Each super individual corresponds to $2^{nl}$ states. The states are listed from up to low according to their better and worse fitness, and the upgrade operation can only better or at least keep super individual(upgrade can only take the lower state to the upper state). In this sense, U should be a low-triangular matrix, i.e.,

$U\text{=}\left(\begin{matrix} U_{11} & & & \\ U_{21} & U_{22} & & \\ \vdots& \vdots& \ddots& \\ U_{2^{l}1} & U_{2^{l}2} & \cdots& U_{2^{l}2^{l}} \end{matrix}\right),$ $U_{11}$is a unit matrix, and $U_{rr},r\geq2$are diagonal matrices with some zero diagonal entries. So, the transition matrix of SG algorithm is

$$\begin{matrix} P^{+}=\left(\begin{matrix} SCMA & & & \\ & SCMA & & \\ & & \ddots& \\ & & & SCMA \end{matrix}\right)\left(\begin{matrix} U_{11} & & & \\ U_{21} & U_{22} & & \\ \vdots& \vdots& \ddots& \\ U_{2^{l}1} & U_{2^{l}2} & \cdots& U_{2^{l}2^{l}} \end{matrix}\right) \\ =\left(\begin{matrix} SCMAU_{11} & & & \\ SCMAU_{21} & SCMAU_{22} & & \\ \vdots& \vdots& \ddots& \\ SCMAU_{2^{l}1} & SCMAU_{2^{i}2} & & SCMAU_{2^{l}2^{l}} \end{matrix}\right), \end{matrix}$$

Here, $SCMAU_{11}=SCMA>0,$it gathers the transition probabilities for states containing a globally optimal super individual, i.e., the global optimal state. In order to utilize LEMMA 1, we can transform the matrix $P^{+}$into the matrix P appearing in LEMMA 1, via letting $\text{R=}\left(\begin{matrix} SCMAU_{21} \\ SCMAU_{31} \\ \vdots\\ SCMAU_{2^{l}1} \end{matrix}\right)\neq0,T=\left(\begin{matrix} SCMAU_{22} & & \\ \vdots& \ddots& \\ SCMAU_{2^{l}2} & \cdots& SCMAU_{2^{l}2^{l}} \end{matrix}\right)\neq0$.

So, we have$\text{P}^{+}\text{=}\left(\begin{matrix} SCMAU_{11} & O \\ R & T \end{matrix}\right)=\left(\begin{matrix} SCMA & O \\ R & T \end{matrix}\right)$. Here, the rows of the matrix SCMA corresponds to all the $2^{nl}$states with global optimum MIC values as super individual, and SCMA is positive and primitive. Based on the LEMMA 1 mentioned above, for any initial state$P^{0}$, there is only limit distribution and the left $2^{nl}$entries are all positive and their sum is 1. The probability of limit non-optimal states is 0, and the probability of limit optimal states is 1. SG can find the optimal MIC value at probability 1, hence we come to the following conclusions:

Proposition 1.5: SG is convergent.

Proposition 1.6: SG is equivalent to the exhaustive algorithm with a sufficient number of iterations.

**Section 3:**

**3.1**

**Table S1: Part table of MIC values from Exhaustive algorithm, SG and ApproxMaxMI** for 1,000,000 relationships of fruit fly gene expression profiles

| var_x | var_y | Exhaustive | SG | ApproxMaxMI |
| --- | --- | --- | --- | --- |
| 18w-RA | 312-RA | 0.406715 | 0.406715 | 0.31127 |
| 18w-RA | 312.a | 0.420448 | 0.420448 | 0.19087 |
| 18w-RA | 5-HT1A-RA | 0.413817 | 0.413817 | 0.19087 |
| 18w-RA | 5-HT1A-RB | 0.413817 | 0.413817 | 0.09328 |
| 18w-RA | 5-HT1B-RA | 0.343579 | 0.343579 | 0.19087 |
| 18w-RA | 5-HT1B-RB | 0.24715 | 0.24715 | 0.19087 |
| 18w-RA | 5-HT1B-RC | 0.540852 | 0.540852 | 0.31127 |
| 18w-RA | 5-HT1B.a | 0.343579 | 0.343579 | 0.1957 |
| 18w-RA | ABCB7-RA | 0.420448 | 0.420448 | 0.19087 |
| 18w-RA | ABCB7-RB | 0.413817 | 0.413817 | 0.19087 |
| 18w-RA | ABCB7-RC | 0.413817 | 0.413817 | 0.1957 |
| 18w-RA | ACXD-RA | 0.413817 | 0.413817 | 0.19087 |
| 18w-RA | APP-BP1-RA | 0.413817 | 0.413817 | 0.19087 |
| 18w-RA | APP-BP1.a | 0.316689 | 0.316689 | 0.19087 |
| 18w-RA | APP-BP1.b | 0.413817 | 0.413817 | 0.19087 |
| 18w-RA | APP-BP1.c | 0.343579 | 0.343579 | 0.19087 |
| 18w-RA | APP-BP1.d | 0.413817 | 0.413817 | 0.1957 |
| 18w-RA | APP-BP1.e | 0.413817 | 0.413817 | 0.19087 |
| 18w-RA | APP-BP1.f | 0.459148 | 0.459148 | 0.45914 |
| 18w-RA | ASPP-RA | 0.979869 | 0.979869 | 0.65485 |
| 18w-RA | ASPP-RB | 0.459148 | 0.459148 | 0.45914 |
| 18w-RA | ASPP.a | 0.540852 | 0.540852 | 0.31127 |
| 18w-RA | AT25722 | 0.24715 | 0.24715 | 0.19087 |
| 18w-RA | AT28206 | 0.617492 | 0.617492 | 0.45914 |
| 18w-RA | AT28783 | 0.24715 | 0.24715 | 0.09328 |
| 18w-RA | ATPsyn-b-RA | 0.654858 | 0.654858 | 0.65485 |
| 18w-RA | ATPsyn-b-RB | 0.476382 | 0.476382 | 0.31127 |
| 18w-RA | ATPsyn-b.a | 0.420448 | 0.420448 | 0.31127 |
| 18w-RA | Aats-ala-m-RA | 0.413817 | 0.413817 | 0.19087 |
| 18w-RA | Aats-leu-RA | 0.343579 | 0.343579 | 0.1957 |
| 18w-RA | Aats-leu.a | 0.413817 | 0.413817 | 0.1957 |
| 18w-RA | Aats-pro-RA | 0.540852 | 0.540852 | 0.31127 |
| 18w-RA | Ack-RA | 0.540852 | 0.540852 | 0.31127 |

……

| var_x | var_y | Exhaustive | | SG | | ApproxMaxMI | |
| --- | --- | --- | --- | --- | --- | --- | --- |
| AT28206 | CG11526-RA | 0.251629 | 0.251629 | | 0.1957 | |  |
| AT28206 | CG11526-RB | 0.168591 | 0.168591 | | 0.09328 | |  |
| AT28206 | CG11529-RA | 0.459148 | 0.459148 | | 0.45914 | |  |
| AT28206 | CG11534-RA | 0.617492 | 0.617492 | | 0.45914 | |  |
| AT28206 | CG11537-MIP21895 | 0.406715 | 0.406715 | | 0.31127 | |  |
| AT28206 | CG11537-RA | 0.343579 | 0.343579 | | 0.1957 | |  |
| AT28206 | CG11537-RB | 0.24715 | 0.24715 | | 0.09328 | |  |
| AT28206 | CG11537-RC | 0.420448 | 0.420448 | | 0.31127 | |  |
| AT28206 | CG11537-RD | 0.285493 | 0.285493 | | 0.09328 | |  |
| AT28206 | CG11537-RE | 0.316689 | 0.316689 | | 0.19087 | |  |
| AT28206 | CG11537.a | 0.654858 | 0.654858 | | 0.65485 | |  |
| AT28206 | CG11537.b | 0.617492 | 0.617492 | | 0.45914 | |  |
| AT28206 | CG11537.c | 0.420448 | 0.420448 | | 0.31127 | |  |
| AT28206 | CG11537.d | 0.420448 | 0.420448 | | 0.19087 | |  |
| AT28206 | CG11537.e | 0.406715 | 0.406715 | | 0.31127 | |  |
| AT28206 | CG11537.f | 0.24715 | 0.24715 | | 0.19087 | |  |
| AT28206 | CG11537.g | 0.420448 | 0.420448 | | 0.1957 | |  |
| AT28206 | CG11560-RA | 0.811278 | 0.811278 | | 0.65485 | |  |
| AT28206 | CG11560.a | 0.343579 | 0.343579 | | 0.1957 | |  |
| AT28206 | CG11570-RA | 0.24715 | 0.24715 | | 0.1957 | |  |
| AT28206 | CG11570.a | 0.413817 | 0.413817 | | 0.1957 | |  |
| AT28206 | CG11570.b | 0.413817 | 0.413817 | | 0.1957 | |  |
| AT28206 | CG11570.c | 0.413817 | 0.413817 | | 0.1957 | |  |
| AT28206 | CG11582-RB | 0.24546 | 0.24546 | | 0.19087 | |  |
| AT28206 | CG11583-RA | 0.343579 | 0.343579 | | 0.1957 | |  |
| AT28206 | CG11586-RA | 0.343579 | 0.343579 | | 0.1957 | |  |
| AT28206 | CG11588-IP09522 | 0.236562 | 0.236562 | | 0.09328 | |  |
| AT28206 | CG11588-RA | 0.311278 | 0.311278 | | 0.31127 | |  |
| AT28206 | CG11593-RB | 0.420448 | 0.420448 | | 0.31127 | |  |
| AT28206 | CG11594-RA | 0.617492 | 0.617492 | | 0.45914 | |  |
| AT28206 | CG11594-RB | 0.316689 | 0.316689 | | 0.19087 | |  |
| AT28206 | CG11594-RC | 0.311278 | 0.311278 | | 0.31127 | |  |
| AT28206 | CG11594.a | 0.459148 | 0.459148 | | 0.45914 | |  |
| AT28206 | CG11597-RA | 0.343579 | 0.343579 | | 0.1957 | |  |
| AT28206 | CG11597-RB | 0.251629 | 0.251629 | | 0.09328 | |  |
| AT28206 | CG11597-RC | 0.24715 | 0.24715 | | 0.09328 | |  |
| AT28206 | CG11652-RA | 0.311278 | 0.311278 | | 0.31127 | |  |
| AT28206 | CG11652-RB | 0.476382 | 0.476382 | | 0.1957 | |  |
| AT28206 | CG11652.a | 0.311278 | 0.311278 | | 0.31127 | |  |
| AT28206 | CG11652.b | 0.420448 | 0.420448 | | 0.31127 | |  |

**……**

**3.2 The simulated annealing and genetic algorithm of SG**

Based on literatures about simulated annealing and genetic algorithm^17-20,25,26^, we designed the following algorithm flow:

**Step 1:** Put in the expression profiles of a gene pair, the number of time points is denoted as *n*. Then, determine n_x_ and n_y_, which are the numbers of bins of *x*-axis and *y*-axis partition for constructing *x*-by-*y* grid, based on the rule: $n_{x}\cdot n_{y}<B(n),B(n)=n^{0.6}.$

**Step 2:** Use parallel computing method to calculate the MIC value by SG. A thread computes optimum fitness S for a pair of n_x_ and n_y_.

**Step 3:** In a thread, for gene expression profile data which usually have less than 50 time points, we set the population size N-size to be 20 by default in our program, the crossover and mutation coefficient to be P_c_, P_m_, and the stop rule, i.e. the max number of consecutive generations of one fitness to be champion q=30. For the simulated annealing algorithm, we choose the proper initial temperature T_0_ and the annealing coefficient α and let the iterative counter k =0.

**Step 4:** In the thread, for the pair of given n_x_ and n_y_, we firstly randomly generate N-size chromosomes as initial population N_0_. Here, the genes are the abscissa (denoted as *xgene*) of vertical lines and the ordinate (denoted as *ygene*) of horizontal lines to form the *x*-by-*y* grid. Calculating the value of objective function $I(x,y)/\log_{2}\min\{n_{x},n_{y}\}$for each of the 20 chromosomes, and get the initial optimum fitness S. P counts the generations a chromosome keeping being the champion.

**Step 5:** Then, we keep the optimum fitness and its corresponding chromosome.

**Step 6:** Crossover and mutate the 20 chromosomes base on the P_c_ and P_m_ respectively, calculate their fitnesses and keep the optimum chromosomes. Note, during the crossover step, we only cross an *xgene* with another *xgene*, a *ygene* with another *ygene,* because *xgene* and *ygene* change independently.

**Step 7:** Perform the simulated annealing, in which the temperature series are T_k+1_= αT_k_，k =k+1. Here, the reproduction operator of Metropolis criteria was used to guarantee the diversity of individuals and to avoid premature convergence, and generate the next generation population N_k+1_.

**Step 8:** After mutation, crossover and simulated annealing, we calculate the fitness values for all the new generation of populations, and let S’ be the maximum fitness of these new populations.

**Step 9:** Compare the new maximum S’ with the old one S, if S’>S, let S=S’ and P=0; otherwise, P=P+1;

**Step 10:** if $P\geq q$(q=30 in our program), stop iterative and the final fitness is the MIC value by SG for the pair of given n_x_ and n_y_; otherwise, return to Step 5;

**Step 11:**  Take the largest fitness value of all threads (i.e., for all possible pairs of n_x_ and n_y_ ) as the MIC value by SG.

**Fig S5: the flow of the SG algorithm**

**3.3**

**Table S2 The comparison of MIC values between SG and ApproxMaxMI**

| 3,000 genes of species | Total number of relationships, i.e. the number of MIC values | The number of relationships which MIC values by SG are larger than those by ApproxMaxMI | The number of relationships which MIC values by SG equal to those by ApproxMaxMI | The number of relationships which MIC values by SG are less than those by ApproxMaxMI |
| --- | --- | --- | --- | --- |
| Yeast | 4,498,500 | 4,348,253 | 28,718 | 121,529 |
| Fruit fly | 4,498,500 | 4,377,054 | 121,392 | 54 |
| locust | 4,498,500 | 4,046,571 | 451,929 | 0 |

**3.4**

**Fig S6** The comparison of MIC values among Exhaustive, SG and ApproxMaxMI algorithms for 1,000,000 pairs of gene expression profiles of fruitfly. The solid line represents the function y=x. Each subfigure includes 1,000,000 points. **
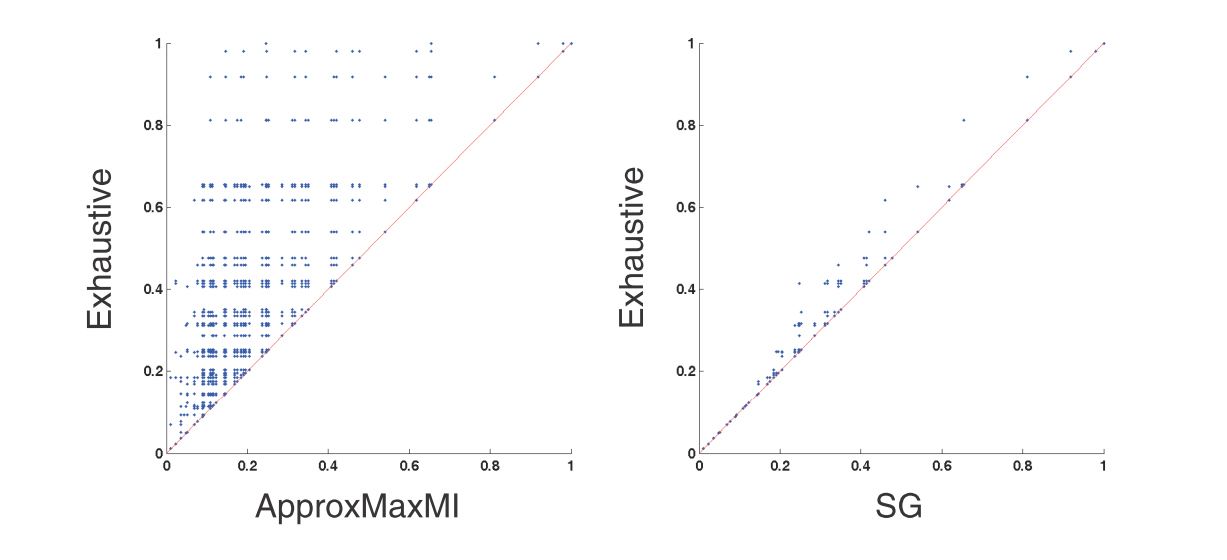
**

**Section 4**

**Table S4**, The 14 formula for Fig. 3

$$\left\{\begin{matrix} y=x \\ y=\frac{1}{2}x \end{matrix}\right.$$

$$x\in[0,2]$$

b:$\left\{\begin{matrix} x=\cos(t) \\ y=\sin(t) \end{matrix}\right.$ $t\in[-\pi,\pi]$

c: $\left\{\begin{matrix} x=\cos(t) \\ y=\sin(t) \end{matrix}\right.$ $\left\{\begin{matrix} y=0 \\ x\in[-1,1] \end{matrix}\right.$,$t\in[-\pi,\pi]$

d: $\left\{\begin{matrix} x_{1}=\cos(t_{1}) \\ y_{1}=\sin(t_{1}) \end{matrix}\right.$ $\left\{\begin{matrix} x_{2}=-0.5+0.5\times\cos(t_{2}) \\ y_{2}=0.5\times\sin(t_{2}) \end{matrix}\right.$ $\left\{\begin{matrix} x_{3}=0.5+0.5\times\cos(t_{3}) \\ y_{3}=0.5\times\sin(t_{3}) \end{matrix}\right.$ $\left\{\begin{matrix} x_{4}=-0.5+0.125\times\cos(t_{4}) \\ y_{4}=0.125\times\sin(t_{4}) \end{matrix}\right.$ $\left\{\begin{matrix} x_{5}=0.5+0.125\times\cos(t_{5}) \\ y_{5}=0.125\times\sin(t_{5}) \end{matrix}\right.$ $t_{1}\in[-\pi,\pi]$,$t_{2}\in[0,\pi]$,$t_{3}\in[-\pi,0]$,$t_{4}\in[-\pi,\pi]$,$t_{5}\in[-\pi,\pi]$

e:$\left\{\begin{matrix} x=e^{-\frac{t}{20}}\times\cos(t) \\ y=e^{-\frac{t}{20}}\times\sin(t) \end{matrix}\right.$ ,$t\in[0,10\pi]$

f:$y=\sin(x)$ ,$x\in[-\pi,\pi]$

g: $y=\tan(x)$,$x\in[-\frac{\pi}{4},\frac{\pi}{4}]$

h: $y=4x^{3}+x^{2}-4x$,$x\in[-1.3,1.1]$

i: $y=4x^{4}+x^{3}-4x^{2}+x$,$x\in[-1.3,1.1]$

j: $y=x^{2}$ ,$x\in[-1,1]$

k: $y=e^{x}$ ,$x\in[-1,1]$

l: $y=\frac{1}{x}$ ,$x\in[-2,2]$

m: $\left\{\begin{matrix} \begin{matrix} \begin{matrix} 200x & if\begin{matrix} & x<\frac{1}{200} \end{matrix} \end{matrix} \\ \begin{matrix} -198x+\frac{199}{100} & if\begin{matrix} & \frac{1}{200}\leq x<\frac{1}{100} \end{matrix} \end{matrix} \end{matrix} \\ \begin{matrix} -\frac{x}{99}+\frac{1}{99} & if\begin{matrix} & x\geq\frac{1}{100} \end{matrix} \end{matrix} \end{matrix}\right.$ $x\in[0,1]$

n: $\left\{\begin{matrix} x=0.5\times(2\cos(t)-\cos(2t)) \\ y=0.5\times(2\sin(t)-\sin(2t)) \end{matrix}\right.$,$t\in[-\pi,\pi]$

**Section 5**

**Table S5 The correlation values measured by 6 algorithms for 56 figures shown in Fig.3.**

|  | SG | Spearman | Pearson | Mutual information  (Covshrink-KPM) | Maximal correlation  (Ace) | energy statistics |
| --- | --- | --- | --- | --- | --- | --- |
| A1 | 0.77317 | 0.8752 | 0.83205 | 6.66E-04 | 0.89193 | 0.31878 |
| A2 | 0.70106 | 0.8753 | 0.83211 | 6.62E-04 | 0.89146 | 0.32015 |
| A3 | 0.68073 | 0.8742 | 0.83183 | 6.67E-04 | 0.88225 | 0.31927 |
| A4 | 0.64555 | 0.8596 | 0.81931 | 6.73E-04 | 0.83729 | 0.31461 |
| B1 | 0.67987 | -6.84E-07 | -4.95E-07 | 1 | 0.99850 | 0.10954 |
| B2 | 0.59828 | 1.48E-04 | -6.90E-05 | 1 | 0.97683 | 0.08584 |
| B3 | 0.54856 | -0.0017 | -0.00324 | 1 | 0.97409 | 0.08466 |
| B4 | 0.45808 | -0.0013 | -4.58E-04 | 1 | 0.89704 | 0.08077 |
| C1 | 0.48853 | -5.20E-04 | -1.38E-07 | 1 | 0.85379 | 0.07611 |
| C2 | 0.37776 | -2.01E-04 | -3.56E-04 | 1 | 0.64299 | 0.05719 |
| C3 | 0.35654 | 0.0043 | 0.00179 | 1 | 0.63178 | 0.05629 |
| C4 | 0.29449 | -0.0038 | 8.02E-04 | 1 | 0.56888 | 0.05330 |
| D1 | 0.41301 | -0.2235 | -0.16972 | 0.0121 | 0.33508 | 0.07422 |
| D2 | 0.35894 | -0.2216 | -0.16744 | 0.0129 | 0.64533 | 0.07343 |
| D3 | 0.29352 | -0.2170 | -0.16762 | 0.0130 | 0.55032 | 0.07264 |
| D4 | 0.21335 | -0.2228 | -0.16743 | 0.0129 | 0.22122 | 0.07261 |
| E1 | 0.19630 | 0.0397 | 0.04984 | 0.1617 | 0.32049 | 0.01992 |
| E2 | 0.14541 | 0.0385 | 0.04852 | 0.1892 | 0.21150 | 0.01953 |
| E3 | 0.10053 | 0.0374 | 0.04782 | 0.1967 | 0.11453 | 0.01972 |
| E4 | 0.06124 | 0.0421 | 0.04838 | 0.1909 | 0.07334 | 0.01870 |
| F1 | 1 | 0.7496 | 0.77992 | 1.89E-04 | 0.99993 | 0.73912 |
| F2 | 0.98915 | 0.7491 | 0.77890 | 2.11E-04 | 0.99936 | 0.73816 |
| F3 | 0.97993 | 0.7491 | 0.77778 | 2.12E-04 | 0.99764 | 0.73719 |
| F4 | 0.94167 | 0.7478 | 0.77535 | 2.17E-04 | 0.99092 | 0.73367 |
| G1 | 1 | 1 | 0.99798 | 4.62E-04 | 1 | 0.34781 |
| G2 | 0.97717 | 0.9992 | 0.99703 | 4.82E-04 | 0.99811 | 0.34831 |
| G3 | 0.94341 | 0.9967 | 0.99470 | 4.87E-04 | 0.99315 | 0.34687 |
| G4 | 0.88402 | 0.9865 | 0.98362 | 4.98E-04 | 0.97400 | 0.34301 |
| H1 | 0.99498 | -0.3764 | -0.38139 | 0.0031 | 0.99972 | 0.37685 |
| H2 | 0.95362 | -0.3613 | -0.36583 | 0.0032 | 0.99543 | 0.37245 |
| H3 | 0.91082 | -0.3632 | -0.36505 | 0.0032 | 0.98311 | 0.37139 |
| H4 | 0.81833 | -0.3630 | -0.36282 | 0.0032 | 0.94042 | 0.36771 |
| I1 | 0.99453 | 0.7552 | 0.76763 | 0.0010 | 0.99973 | 0.50100 |
| I2 | 0.96221 | 0.7499 | 0.76011 | 9.86E-04 | 0.99488 | 0.50048 |
| I3 | 0.93468 | 0.7497 | 0.75894 | 9.91E-04 | 0.98168 | 0.49904 |
| I4 | 0.87316 | 0.7502 | 0.75646 | 9.96E-04 | 0.94700 | 0.49590 |
| J1 | 0.99998 | -3.45E-17 | 0.00194 | 1 | 1 | 0.14918 |
| J2 | 0.95457 | -0.0015 | 5.41E-04 | 1 | 0.99541 | 0.14969 |
| J3 | 0.90516 | -0.0016 | 0.00308 | 1 | 0.98194 | 0.14782 |
| J4 | 0.81180 | 0.0060 | 0.00814 | 1 | 0.93859 | 0.14372 |
| K1 | 1 | 1 | 0.96908 | 4.85E-04 | 1 | 0.43708 |
| K2 | 0.97878 | 0.9991 | 0.96827 | 4.85E-04 | 0.99857 | 0.43863 |
| K3 | 0.96013 | 0.9968 | 0.96710 | 4.88E-04 | 0.99548 | 0.43768 |
| K4 | 0.91462 | 0.9881 | 0.96009 | 4.92E-04 | 0.98341 | 0.43405 |
| L1 | 1 | 0.5000 | 0.04270 | 9.91E-32 | 0.93659 | 0.78088 |
| L2 | 0.99451 | 0.5003 | 0.09502 | 9.03E-06 | 0.95427 | 0.77877 |
| L3 | 0.99068 | 0.5010 | 0.09609 | 2.77E-05 | 0.94434 | 0.77728 |
| L4 | 0.97569 | 0.5033 | 0.09543 | 1.10E-04 | 0.95417 | 0.77226 |
| M1 | 1 | -0.7729 | -0.59684 | 7.51E-04 | 0.99971 | 0.15981 |
| M2 | 0.86079 | -0.7378 | -0.60173 | 6.70E-04 | 0.86705 | 0.16261 |
| M3 | 0.76347 | -0.7130 | -0.59840 | 6.97E-04 | 0.78722 | 0.15800 |
| M4 | 0.62061 | -0.6561 | -0.57871 | 7.59E-04 | 0.65324 | 0.14857 |
| N1 | 0.67436 | -3.77E-04 | 8.56E-08 | 1 | 0.99342 | 0.15119 |
| N2 | 0.63895 | -2.63E-04 | 9.34E-06 | 1 | 0.97827 | 0.15049 |
| N3 | 0.58835 | -0.0026 | -0.00126 | 1 | 0.97718 | 0.14841 |
| N4 | 0.50286 | -0.0083 | -9.50E-04 | 1 | 0.92069 | 0.14526 |

**Section 6**

**Table S6** Part table of MIC values by SG and MIC values by GA without simulation annealing. The negative values of Difference indicate the advantage of SG over GA in optimizing MIC calculation.

| Var x | Var Y | MIC by SG | MIC by GA | Difference |
| --- | --- | --- | --- | --- |
| data1 | data2 | 0.194281 | 0.212589 | 0.018308 |
| data1 | data3 | 0.158709 | 0.175158 | 0.016449 |
| data1 | data4 | 0.157014 | 0.148607 | -0.00841 |
| data1 | data5 | 0.167897 | 0.157736 | -0.01016 |
| data1 | data6 | 0.145057 | 0.12615 | -0.01891 |
| data1 | data7 | 0.140581 | 0.132151 | -0.00843 |
| data1 | data8 | 0.141673 | 0.161908 | 0.020235 |
| data1 | data9 | 0.149101 | 0.17611 | 0.027009 |
| data1 | data10 | 0.130215 | 0.131054 | 0.000839 |
| data1 | data11 | 0.150798 | 0.138638 | -0.01216 |
| data1 | data12 | 0.154424 | 0.136834 | -0.01759 |
| data1 | data13 | 0.187389 | 0.174562 | -0.01283 |
| data1 | data14 | 0.13493 | 0.121375 | -0.01356 |
| data1 | data15 | 0.135212 | 0.13611 | 0.000898 |
| data1 | data16 | 0.186856 | 0.143663 | -0.04319 |
| data1 | data17 | 0.122761 | 0.122408 | -0.00035 |
| data1 | data18 | 0.137114 | 0.147586 | 0.010472 |
| data1 | data19 | 0.158919 | 0.154968 | -0.00395 |
| data1 | data20 | 0.138193 | 0.132358 | -0.00584 |
| data1 | data21 | 0.148449 | 0.144383 | -0.00407 |
| data1 | data22 | 0.172261 | 0.133424 | -0.03884 |
| data1 | data23 | 0.162638 | 0.164989 | 0.002351 |
| data1 | data24 | 0.16312 | 0.143756 | -0.01936 |
| data1 | data25 | 0.137748 | 0.118054 | -0.01969 |
| data1 | data26 | 0.158653 | 0.143476 | -0.01518 |
| data1 | data27 | 0.18643 | 0.157358 | -0.02907 |
| data1 | data28 | 0.161984 | 0.153609 | -0.00837 |
| data1 | data29 | 0.137941 | 0.129726 | -0.00822 |
| data1 | data30 | 0.146896 | 0.153031 | 0.006135 |
| data1 | data31 | 0.142284 | 0.128321 | -0.01396 |
| data1 | Data32 | 0.12978 | 0.122186 | -0.00759 |
| Data1 | Data33 | 0.166398 | 0.153894 | -0.0125 |
| Data1 | Data34 | 0.173088 | 0.166321 | -0.00677 |
| Data1 | Data35 | 0.136966 | 0.142678 | 0.005712 |

.......

**Section 7**

**Fig S7** Procedure searching for an optimal value of B(n).


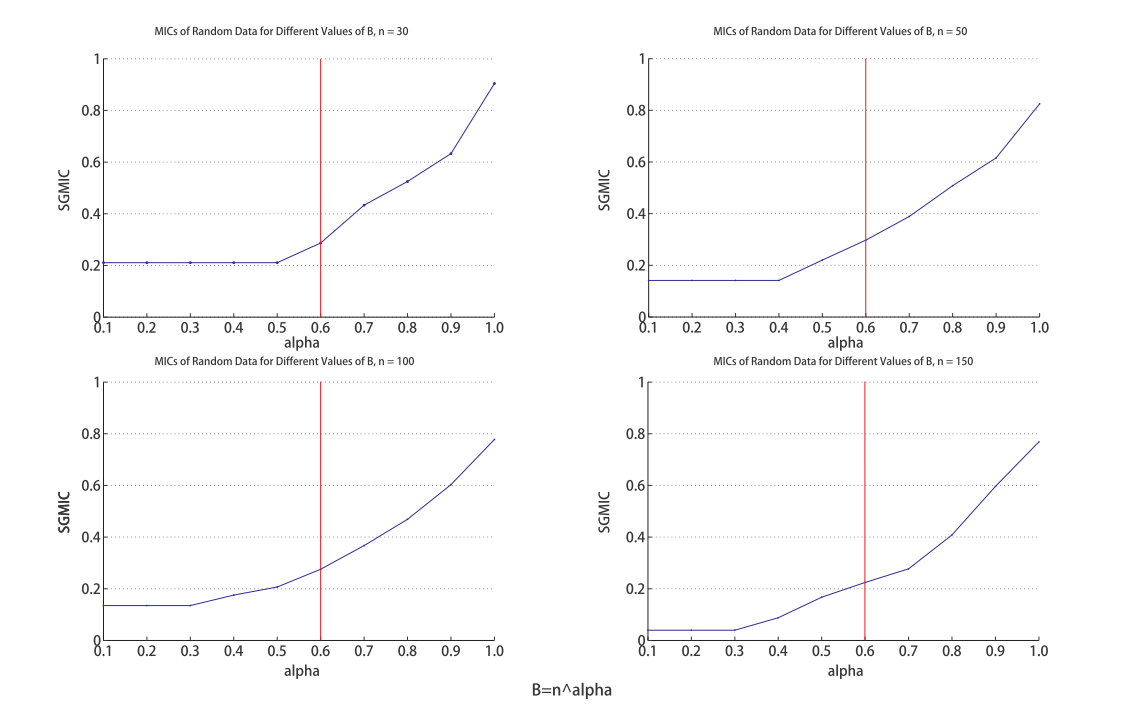


**Additional Reference of SM:**

25. Rudolph, G. Convergence Analysis of Canonical Genetic Algorithms. *IEEE T. Neural Networ.*, **5**, 96-101 (1994).

26. Eiben A.E., Aarts E.H.L., Hee,K.M.V. Global Convergence of Genetic Algorithms: A Markov Chain Analysis. *Lect. Notes in Comput. Sc.,* **496**, 3-12 (1991).
